# Case of Contusion, Followed by a Remarkable Retention of
Urine: Communicated to Dr. Miller

**Published:** 1810-11

**Authors:** E. F. R. Smith

**Affiliations:** Physician in the City of New-York


### *Case of Contusion, followed by a remarkable retention of Urine: Communicated to Dr.* Miller, *by Dr.* E. F. R. Smith, *Physician in the City of New-York.*

LYDIA ——, a servant maid, slipped on the ice and struck her pudenda against the edge of a board. Great inflammation succeeded, and an immediate suppression of urine took place, although in the act of going out for the purpose of voiding it. The accident occurred on the 25th of January, and on the 27th I first saw the patient. She complained of no pain, except soreness of the parts ; had rested well, and was without symptoms of fever. She said she had passed no urine since the injury, except once or twice, about a tea-cup-full, bloody, and accompanied with great pain. Some of it I inspected, and found small coagula of blood, mixed with the water. Upon examination, for the purpose of introducing the catheter, the labia and small neighbouring glands were found so much tumefied and deranged, that the attempt to afford relief, in this way, proved unsuccessful. Ordered fomentations, emollient applications and rest ; 28th, symptoms the same. Ordered a brisk cathartic. 29th, medicine operated well. No alteration of symptoms, except a little tension of the pulse. Took from the arm ten ounces of blood ; and in the afternoon succeeded in introducing the catheter, but without obtaining more than a spoonful of water. The singularity of the case now fully appeared. Four days had elapsed since the accident, when the bladder must have been full ; and in that time she had passed only about eight ounces of water, at the end of which the bladder was found empty. There was no reason to

 suspect suspect an accumulation in the kidneys or ureters, else there would have been pain, and other specific symptoms; in short, it was obvious, there had been no urine secreted. Her appetite had been indulged, and she had not refrained from drinks. On the 30th, my colleague, Dr. Robson, was called in consultation, who agreed with me in opinion, that this was an extraordinary case of suspension of the secretion of urine. Symptoms same as yesterday. Passed no water, no tumefaction of abdomen, no uneasiness of any kind. Ordered a powder consisting of nitrate of potash, grs. x. submuriate of mercury, grs. ij. repeated three times a day. Saturnine application to the parts. 31st; inflammation and sense of soreness much abated; great anxiety; tense pulse; restless during night; passed no water; stomach rejected last powder; no alvine evacuation since 29th. Ordered the common enema to be administered immediately, which afforded much relief. Substituted for the powder, tincture of digitalis, twenty drops, three times a day. Directed a cathartic of calomel and jalap to-morrow morning. February 1st, complains of pain across the loins; bladder empty, but no passage of water; pulse small and tense; little appetite; cathartic, assisted by injection, operated well. Ordered tincture of digitalis, increased two drops at every dose, and a calomel pill of one grain, with a little opium, three times a day. Drink plentifully of flax-seed tea. 2nd. Pain in the loins subsided. Pulse slow and more full; passed half a tea-cup-full of transparent urine; medicines continued, with an injection daily. 3d. Gums sore; pulse slow and wirey; little pain in the lumbar region; no passage of water; continued pills once a day, tincture as before. 4th. Very uneasy, with pain about the loins and region of the bladder, increased by pressure upon the abdomen. Pulse more hurried. Introduced the catheter and drew off a pint of limpid urine, which gave much relief. During the two following days the patient continued much as the last. Drew off, each day, half a pint of clear urine. On the 6th, observed some disorder of the stomach; suppose, in consequence of the digitalis, of which the last dose was 35 drops. Directed the quantity to be diminished 5 drops at each dose, down to 10, which continue. After this time the patient was enabled to pass her urine without assistance; at first, in small quantities, but gradually increasing. She continued the use of digitalis and the calomel pill till the 24th, when she was discharged cured.

In the history of this case, the most striking circumstance is, that of the suspended secretion of urine. During the first eight days of the disease, the whole quantity of water secreted secreted did not exceed half a pint, and in the latter four of these days, not a drop was made. That such an effect should arise from so slight a cause as an external injury to the pudenda, appears very extraordinary. There was no symptom of inflammation extending to the kidneys or even to the bladder; nor was there reason to suspect the existence of calculi in the urinary passages. Indeed, there is no possibility of accounting for the suppression of this necessary evacuation, but by supposing the function of the kidneys to have been entirely suspended. The appearance of small coagula of blood in the first passage of urine, I suppose to be owing to the injury of the external parts; as likewise the pain, upon attempting to make water, while the inflammation lasted.

I find no cases upon record in all respects similar to this. Indeed, I believe the complete suppression of urine to be a very uncommon disease. Mr. Hey, in his "Practical Observations in Surgery," mentions that he has seen but a few cases of the *ischuria renalis*, or complete suppression of the secretion of urine by the kidneys. The disease proved fatal in all his patients, except one, in whom it was brought on by the effect of lead taken into the body by working in a pottery. It subsisted three days, during a violent attack of the colica pictonum, and was then removed, together with the original disease.

With regard to the treatment of my patient, I have very little to add. In this we were almost without a guide; and the existing symptoms formed our only indications. I would remark, however, that notwithstanding a free exhibition of the digitalis, the kidneys did not show a returning disposition to their office, till the system began to be affected by the mercury. They were both continued with but little inconvenience, till the patient was discharged.

